# No Time to Bleed! Ultrasound‐Guided Management of a Pleural Biopsy Complication: A Case Report

**DOI:** 10.1002/rcr2.70595

**Published:** 2026-05-18

**Authors:** Daniel Piamonti, Francesca Stirpe, Noemi Calabrò, Erminio Carriero, Daria Romaniello, Raffaele Sglavo, Enrico Giarnieri, Elisabetta Carico, Pasquale Pignatelli, Matteo Bonini, Alberto Ricci

**Affiliations:** ^1^ Department of Clinical, Internal, Anesthesiologic and Cardiovascular Sciences Sapienza University of Rome Rome Italy; ^2^ Department of Cardiovascular and Respiratory Sciences, S Andrea Hospital Sapienza University of Rome Rome Italy; ^3^ Cytopathology Unit, Department of Clinical and Molecular Medicine, Sant'Andrea Hospital Sapienza University of Rome Rome Italy; ^4^ Department of Public Health and Infectious Diseases Sapienza University of Rome Rome Italy

**Keywords:** interventional pulmonology, malignant pleural disease, malignant pleural effusion, pleural haemorrhage, ultrasound‐guided biopsy

## Abstract

Malignant pleural disease is a clinical condition of growing health importance, often presenting as pleural thickening and/or malignant pleural effusion. Rapid and accurate diagnosis is crucial to guide treatment decisions. In most cases, pleural biopsy is necessary: thoracoscopy, ultrasound‐ and CT‐guided biopsies all offer high accuracy and good safety. The use of ultrasound has revolutionised procedural approaches, increasing safety thanks to its good sensitivity and very high specificity, allowing direct and immediate visualisation of complications such as pneumothorax or haemorrhage. In this case report, we describe how intrapleural adrenaline instillation successfully controlled pleural haemorrhage, which was identified as a complication of ultrasound‐guided pleural biopsy thanks to real‐time ultrasound and colour Doppler monitoring of the procedure. Adrenaline was administered under ultrasound guidance directly into the vascular structure presumed to be the source of the bleeding. This technique can be considered an effective and minimally invasive approach for managing bleeding complications of ultrasound‐guided pleural biopsy, even though larger studies are needed to validate efficacy and safety of this approach.

## Graphical abstract

Ultrasound‐guided biopsies allow direct visualisation of target lesions, improve sampling site selection and have shown a significant reduction in major complications. Our case shows the efficacy of intrapleural adrenaline instillation in the management of pleural bleeding due to ultrasound‐guided pleural biopsy.

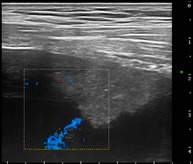

## 1 Introduction

Malignant pleural disease (MPD) is a clinical condition of growing health importance, often presenting as pleural thickening and/or malignant pleural effusion (MPE), both associated with considerable symptom burden and poor prognosis. MPE can cause dyspnea, cough and chest pain, and it is linked to a median survival of only a few months, despite novel therapies. Rapid and accurate diagnosis is crucial to guide treatment decisions and optimise quality of life. In most cases, pleural biopsy is necessary, because of non‐diriment pleural effusion cytology, or to have enough tissue sample to perform molecular biology. Blind techniques are now rarely used, given their lower diagnostic yield and increased risk of complications [1]. Thoracoscopy, ultrasound‐ and CT‐guided biopsies all offer high accuracy and good safety. Ultrasound guidance allows for direct visualisation of lesions, improved sampling site selection and a significant reduction in major complications. It is quickly available and minimally invasive, avoiding radiation exposure and allowing real‐time needle monitoring. The choice depends primarily on available resources and operator's experience [2]. The use of ultrasound has revolutionised procedural approaches, increasing safety thanks to its good sensitivity and very high specificity, allowing direct and immediate visualisation of complications such as pneumothorax or haemorrhage.

## 2 Case Report

**FIGURE 1:**
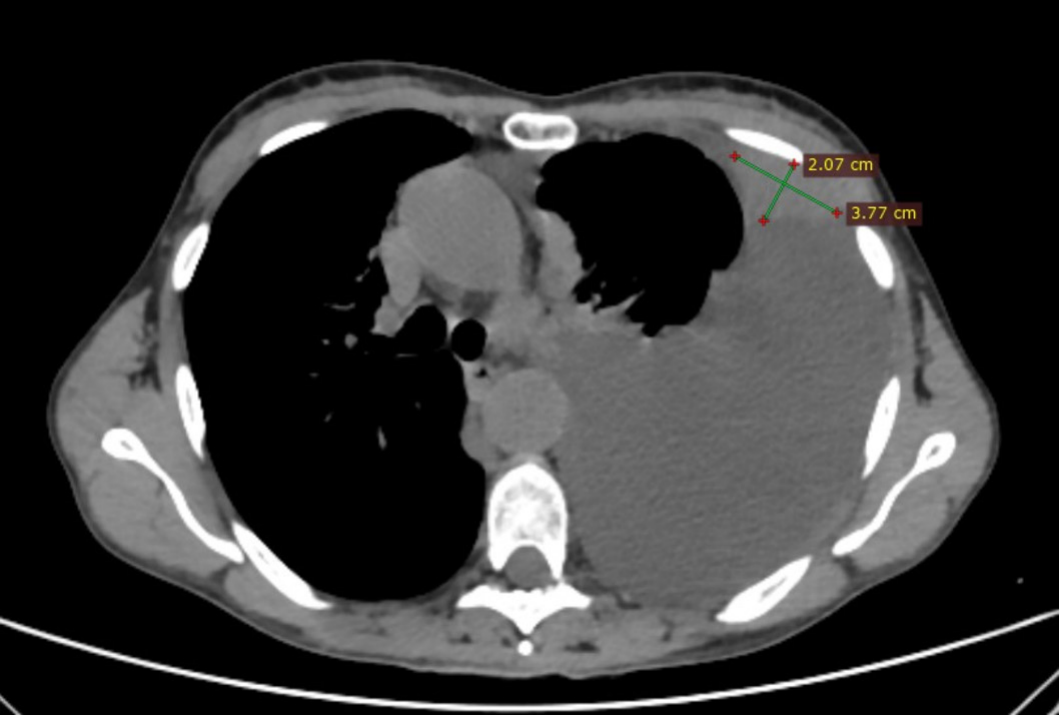
Axial view of a High‐Resolution chest CT scan in the mediastinal window showing a massive left pleural effusion and the largest pleural nodule on the anterior parietal pleura, which underwent ultrasound‐guided percutaneous biopsy.

**FIGURE 2:**
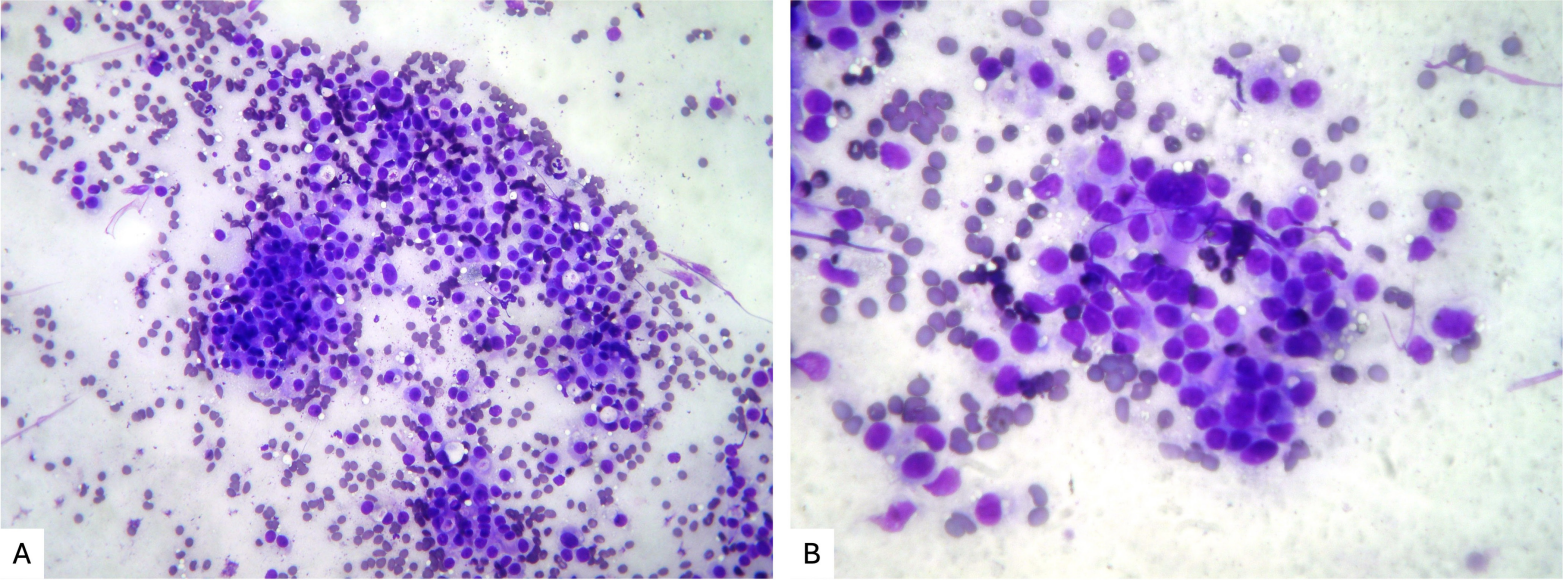
Rapid on‐site evaluation (ROSE) smear from the biopsy sample. 10× (A) and 20× magnification (B) of the smear, showing isolated cells and group of cells with monomorphic characteristics, highly suggestive of neoplastic cells. Also, the specimen seems adequate in quantity for eventual genetic testing.

**FIGURE 3:**
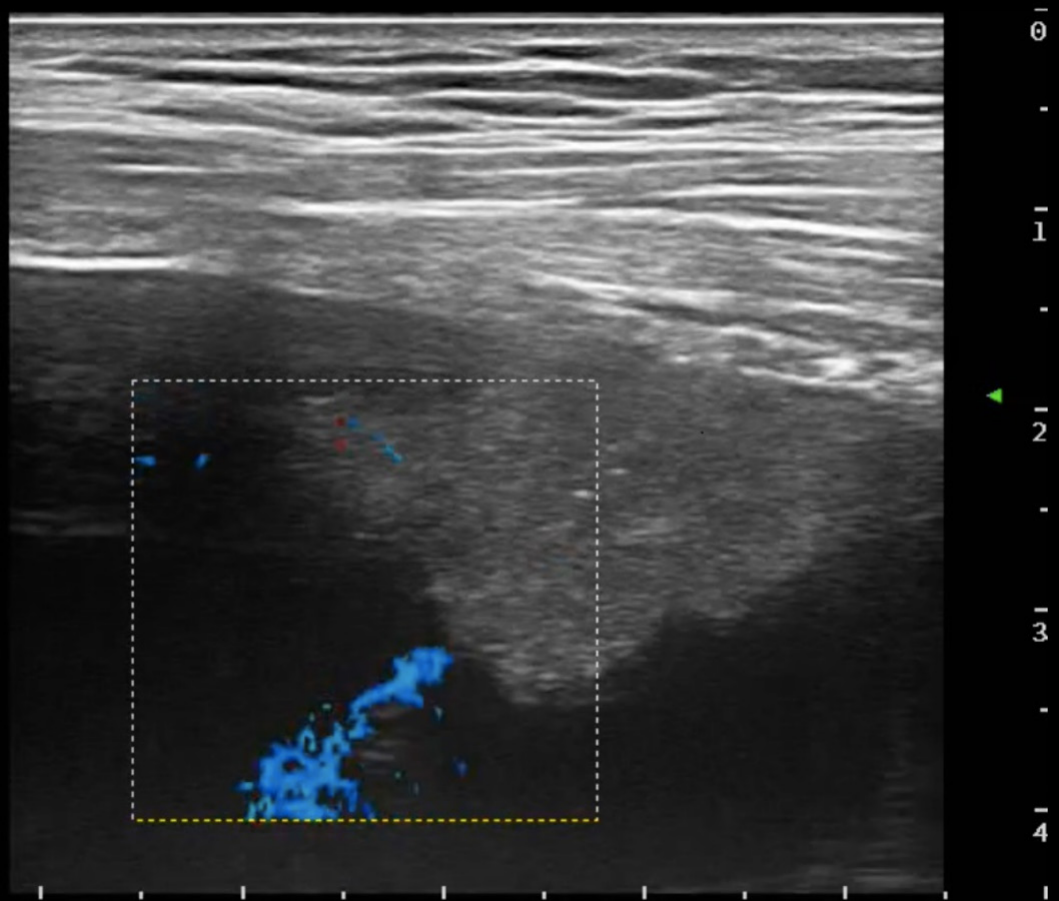
Colour Doppler ultrasound findings suggestive of active intrapleural bleeding from the biopsy site.

A 62‐year‐old woman presented to the emergency room of our institution with persistent dyspnea at rest and cough in the past week. She denied previous respiratory diseases. Blood tests were normal, and a chest X‐Ray showed a large left pleural effusion with mild lateral deviation of the mediastinal structures. Bed‐side chest ultrasonography was performed showing a simple, anechoic massive left pleural effusion. Ultrasound linear probe findings were suggestive of a nodular, solid and hypoechoic lesion of the parietal pleura, highly suspect for MPD. Colour Doppler ultrasound was able to identify vascular structures within the lesion. A high‐resolution chest CT scan confirmed the massive left pleural effusion and the multiple solid pleural nodules (the largest measuring 3.8 cm) (Figure 1). A 12 Fr pleural drain was placed allowing drainage of 1500 mL of pleural fluid of sierous‐haematic aspect. Afterwards, an in‐plane, ultrasound‐guided, percutaneous biopsy of the largest pleural lesions was performed by using an 18 gauge semi‐automatic Tru‐Cut biopsy needle. After the first biopsy puncture, the bioptic sample was immediately passed on a glass slide for rapid on‐site evaluation (ROSE), confirming the presence of an adequate quantity of atypical cells for diagnostic purposes (Figure 2). The remaining part of the sample was fixed in formalin for histopathological analysis. Ultrasound monitoring immediately showed the appearance of echogenic material projecting into the pleural space from the biopsy site, suggesting an active intrapleural bleeding, which was confirmed by colour Doppler ultrasonography (Figure 3). Further biopsies were avoided, ice was applied on the patient's chest, but the presence of residual pleural fluid in the surroundings of the biopsied lesion acted as a site of minor resistance, facilitating bleeding. Adrenaline 0.5 mg diluted in 5 mL of 0.9% saline solution was injected by re‐entering the biopsy site under ultrasound guidance, and applying it directly to the vascular structure presumed to be the source of the bleeding, leading to a rapid resolution. Video 1 resumes the main passages of the whole procedure. Ultrasound monitoring was performed for the following 2 h without evidence of active bleeding, along with a complete blood count 4 h later, which excluded anaemia. The pleural drain, already in place, was opened, draining additional fluid with no evidence of variations in pleural fluid aspect. No other complications were reported during or after the procedure and in the following days there was no evidence of anaemia. A diagnosis of lung adenocarcinoma was obtained and the histological specimens were adequate for molecular biology tests. The patient was then referred to the thoracic surgeon for VATS talc pleurodesis and is undergoing chemotherapy at the moment.

**VIDEO 1:** Main passages of the whole procedure. Video content can be viewed at https://onlinelibrary.wiley.com/doi/10.1002/rcr2.70595.

## 3 Discussion

In this case report, we describe how intrapleural adrenaline instillation successfully controlled pleural haemorrhage as a complication of ultrasound‐guided pleural biopsy. Thoracic ultrasound (TUS) can identify active bleeding because blood appears echogenic and can appear as a turbulent flow in the case of active effusions. TUS may also be valuable in the management of haemorrhage through continuous monitoring [3]. The most recent international guidelines [1, 4] strongly recommend the use of ultrasounds as the standard for pleural biopsy and for intra‐procedural monitoring, the use of safe local anaesthetics, and the availability of tools to control complications. Haemorrhage remains a rare but potentially serious complication. Conventional management typically includes observation, supportive measures and occasionally interventional radiology or surgical intervention. Local compression, ice appliance and systemic antifibrinolytics are also often used, but there are limited reports of intrapleural pharmacological interventions for bleeding control. Adrenaline is well established in airway and gastrointestinal bleeding management, but, to our knowledge, its direct, ultrasound‐guided, intrapleural use for pleural haemorrhage has not been reported, even though its co‐administration with Lidocaine for local anaesthesia is commonly used in pleural procedures [5]. This approach provided a minimally invasive, rapidly effective alternative to more aggressive interventions, in a context where medical thoracoscopy was not available.

In conclusion, ultrasound‐guided intrapleural adrenaline instillation can be considered an effective approach for managing bleeding complications of ultrasound‐guided pleural biopsy. This strategy, supported by real‐time colour Doppler ultrasound, may expand the therapeutic armamentarium for procedural safety in pleural interventions. Larger studies are needed to validate efficacy and safety of this approach. Limitations include the anecdotal nature of a single case, and the need for further evaluation of dosing, safety and efficacy in broader patient cohorts.

## Author Contributions

Daniel Piamonti had full access to all of the data in the study and takes responsibility for the integrity and the accuracy of the data presented. All other authors (Francesca Stirpe, Noemi Calabrò, Erminio Carriero, Daria Romaniello, Raffaele Sglavo, Enrico Giarnieri, Pasquale Pignatelli and Alberto Ricci) contributed substantially to the study design, data collection and interpretation, and the writing of the manuscript.

## Funding

The authors have nothing to report.

## Consent

The authors declare that written informed consent was obtained for the publication of this manuscript and accompanying images and attest that the form used to obtain consent from the patient complies with the Journal requirements as outlined in the author guidelines.

## Conflicts of Interest

The authors declare no conflicts of interest.

## Data Availability

The data that support the findings of this study are available on request from the corresponding author. The data are not publicly available due to privacy or ethical restrictions.

## References

[1] D. J. Feller‐Kopman , C. B. Reddy , M. M. DeCamp , et al., “Management of Malignant Pleural Effusions: An Official ATS/STS/STR Clinical Practice Guideline,” American Journal of Respiratory and Critical Care Medicine 198 (2018): 839–849. 30272503 10.1164/rccm.201807-1415ST

[2] F. Mei , M. Bonifazi , M. Rota , et al., “Diagnostic Yield and Safety of Image‐Guided Pleural Biopsy: A Systematic Review and Meta‐Analysis,” Respiration 100 (2021): 77, 10.1159/000511626–87. 33373985 10.1159/000511626

[3] P. Impellizzeri , G. Levi , D. Piamonti , C. Locorotondo , and G. P. Marchetti , “The Smoke Swirl,” Chest 166 (2024): e47–e49. 39122307 10.1016/j.chest.2024.02.041

[4] M. E. Roberts , N. M. Rahman , N. A. Maskell , et al., “British Thoracic Society Guideline for Pleural Disease,” Thorax 78 (2023): S1–S42. 10.1136/thorax-2022-219784 37433578

[5] C. A. Mounsey , I. R. Mechie , D. N. Addala , et al., “Local Anesthetic Use in Pleural Procedures: Time to Reconsider the Guidelines?,” Chest 168, no. 3 (2025): 839–842, 10.1016/j.chest.2025.04.010. 40254151 PMC12489358

